# Editorial: Ca^2+^ Signaling and Heart Rhythm

**DOI:** 10.3389/fphys.2015.00423

**Published:** 2016-01-11

**Authors:** Christopher L.-H. Huang, R. John Solaro, Yunbo Ke, Ming Lei

**Affiliations:** ^1^Physiological Laboratory, Department of Biochemistry, University of CambridgeCambridge, UK; ^2^Department of Physiology and Biophysics, University of Illinois at ChicagoChicago, USA; ^3^Department of Pharmacology, University of OxfordOxford, UK

**Keywords:** calcium, sino-atrial node, atrial arrhythmia, Ca^2+^ clock, Ca^2+^ channels

Ca^2+^ is a strategic intracellular second messenger regulating multifarious cardiac cellular processes. This Frontiers issue on Ca^2+^ signaling and cardiac rhythm first focuses on the spontaneous membrane depolarization triggering action potential (AP) pacing by sino-atrial node (SAN) cells. These drive normal rhythmic atrial followed by ventricular depolarization initiating effective systolic contraction (Mangoni and Nargeot, 2008). Classic pharmacological and immunological localization studies had implicated sarcoplasmic reticular (SR)-mediated Ca^2+^ storage and release (Rigg and Terrar, 1996) involving ryanodine receptor (RyR2)-Ca^2+^ release channels (Rigg et al., 2000) as necessary components in an adrenergically-responsive, complex, Ca^2+^-dependent, sino-atrial pacing process. Subsequent confocal imaging demonstrated spontaneous, precisely timed, rhythmic, local, submembrane, SR Ca^2+^ release events (Bogdanov et al., 2001; Vinogradova et al., 2004; Lakatta et al., 2010). Were these to activate Na^+^-Ca^2+^ exchange current, I_NCX_, the resulting depolarization could trigger surface inward L-type Ca^2+^ currents, I_Ca_, thereby initiating AP firing (Vinogradova et al., 2002). SAN cells possessed high basal cAMP and phosphokinase A-dependent phosphorylation levels (Vinogradova et al., 2006) that could ensure RyR2-mediated Ca^2+^ release activity (Yang et al., 2002) at the requisite frequencies (Vinogradova et al., 2002, 2006). The resulting [Ca^2+^] (to >100 nM) increases produced the expected I_NCX_ changes (Bogdanov et al., 2001) besides additionally activating strategic enzymes, particularly calcium/calmodulin-dependent protein kinase II (CaMKII). Hyperpolarization-activated cyclic nucleotide-gated (HCN) channels carrying I_f_ likely also importantly contribute to this process: *Hcn4*-/- and *Hcn4*-R669Q/R669Q mouse embryos were bradycardic with 75–90% reduced I_f_ before eventual lethality (Stieber et al., 2003; Chandra et al., 2006; Harzheim et al., 2008); tamoxiphen-inducible adult hearts showed ~70% reduced I_f_ and progressive ≤ 50% reductions in, nevertheless persistent, SAN pacing, compromising its responses to isoproterenol challenge (Sohal et al., 2001; Baruscotti et al., 2011).

The present articles first complete necessary conditions for such a Ca^2+^-mediated pacing system (Vinogradova et al., 2000; Bogdanov et al., 2001; Sanders et al., 2006; Maltsev and Lakatta, 2007) to exist. They explore recent evidence implicating I_NCX_, combined with delayed rectifier K^+^ current deactivation, in the pacemaker depolarization triggering I_Ca_ (Capel and Terrar). Furthermore, intracellular [Ca^2+^] proved instrumental in determining pacing rates: 1,2-bis(o-aminophenoxy)ethane-N,N,N′,N′-tetraacetic acid (BAPTA) dose-dependently slowed, ultimately abolishing, AP firing in isolated guinea-pig SAN myocytes (Capel and Terrar). Involvement of I_Ca_ in both SAN pacing and atrioventricular conduction was indicated in mice homozygously lacking L-type, Cav1.3, or T-type, Cav3.1, channels normally expressed in mouse, rabbit and human pacemaker tissue (Mesirca et al.). Volume and pressure overload-induced heart failure in rabbit SAN cells markedly influenced both Ca^2+^ transients and pacemaker activity (Verkerk et al.). Finally, the hypothesis generated schemes amenable to quantitative modeling and reconstruction (Yaniv et al.).

The articles then explore further ion channel mechanisms possibly contributing to this regulation. TRPC3 channels mediating Ca^2+^ entry are up-regulated in clinical and experimental atrial fibrillation (AF), and are implicated in SAN dysfunction and atrioventricular block (Yanni et al., 2011; Harada et al., 2012; Sabourin et al., 2012). Ju et al. report that the *Trpc3*-/- variant rescued pacing-induced AF in angiotensin II-treated mice (Ju et al.). Similarly, intracellular Ca^2+^ store depletion increased Ca^2+^ entry in isolated firing mouse SAN pacemaker cells, findings reduced by store-operated Ca^2+^ entry (SOCE) blockers. SAN pacemaker cells further expressed the endoplasmic reticular, Ca^2+^-sensing, stromal interacting molecules (STIM) and surface membrane Orai1 channels likely involved in SOCE. Ca^2+^ store depletion redistributed STIM1 to the cell periphery increasing STIM1-Ora1 co-localization (Liu et al.).

SAN and surrounding atrial tissue form a SAN-atrial pacemaker complex. SAN disorders accordingly can produce re-entrant substrate causing AF in addition to bradycardic, sinus node, dysfunction (Nattel et al., 2007). Altered intracellular Ca^2+^ transients and diastolic SR Ca^2+^ release appear to be important AF triggers in murine hearts (Zhang et al., 2009, 2010). Ai explores possible interactions between key Ca^2+^ handling proteins in such arrhythmia. These include RyR2, phospholamban, L-type Ca^2+^ channels (Cav1.2) (Schulman et al., 1992), and possible actions upon these of the intrinsic stress-related family of mitogen-activated protein kinase (MAPK) cascades including c-Jun N-terminal kinase, extracellular signal-regulated kinases, and p38 MAPKs whose activity alters in aging and failing hearts (Ai).

Further articles bear upon modulatory influences upon the complex of Ca^2+^ signaling pathways. Thus, SR Ca^2+^ uptake mechanisms proved affected by p21-activated kinase (Pak1) deficiency, previously identified with hypertrophic ventricular remodeling in heart failure, through altered post-transcriptional activity of key Ca^2+^-handling proteins, particularly SR Ca^2+^-ATPase (Wang et al.). Similarly, altered protein phosphatase 2A expression and activity, likely acting downstream of Pak1, may compromise responses to β-adrenergic stimulation with implications for arrhythmia and cardiac failure (Lei et al.). Finally, membrane protein regulation, trafficking and recycling are fundamental to all cellular physiological processes including those involving Ca^2+^ homeostasis. This prompted review of a particular, endosome-based, trafficking process, involving endocytic C-terminal Eps15 homology domain-containing regulatory proteins (Curran et al.).

Ultimately, quantitative analysis of Ca^2+^-mediated modulatory effects on cardiac function as a whole must extend such molecular and cellular analysis from Ca^2+^ homeostatic to contractile function in entire cardiac chambers (cf. Adeniran et al., 2013; Davies et al., 2014). This reconstruction will require further, more quantitative, data on the processes involved. Nevertheless, one such article succeeds in integrating abnormal Ca^2+^ homeostasis, ion channel and structural remodeling with ventricular electro-mechanical dynamics in the clinical problem of heart failure with preserved ejection fraction. It emerges with testable predictions of reduced systolic Ca^2+^ and therefore systolic force, but increased diastolic Ca^2+^ and therefore residual diastolic force, despite conserved ejection fraction, particularly at increased heart rates (Adeniran et al.). Simulations of this kind offer openings into more detailed and quantitative studies of sino-atrial and atrial intricacies.

Explorations of the kind described in this series of articles thus contribute to development of a systems basis for sinus node disorder (SND), atrial arrhythmia, and their translational consequences (Nattel, 2002). SND is the commonest clinical indication requiring pacemaker implantation. AF, for which available treatment is limited (Kannel and Benjamin, 2009), is a major contributor to cardiovascular morbidity and mortality, particularly in aging human populations (Juhaszova et al., 2005).

## Author contributions

CH, Drafting and planning of the editorial, response to editors report; RS, Re-reading and rewriting of the editorial; YK, Re-reading and checking of the editorial; and ML, Final corrections and rewordings, response to editors report.

### Conflict of interest statement

The authors declare that the research was conducted in the absence of any commercial or financial relationships that could be construed as a potential conflict of interest.
